# Giant Valley Coherence at Room Temperature in 3R WS_2_ with Broken Inversion Symmetry

**DOI:** 10.34133/2019/6494565

**Published:** 2019-10-13

**Authors:** Luojun Du, Jian Tang, Jing Liang, Mengzhou Liao, Zhiyan Jia, Qinghua Zhang, Yanchong Zhao, Rong Yang, Dongxia Shi, Lin Gu, Jianyong Xiang, Kaihui Liu, Zhipei Sun, Guangyu Zhang

**Affiliations:** ^1^Beijing National Laboratory for Condensed Matter Physics and Institute of Physics, Chinese Academy of Sciences, Beijing 100190, China; ^2^Department of Electronics and Nanoengineering, Aalto University, Tietotie 3, FI-02150, Finland; ^3^State Key Laboratory for Mesoscopic Physics, Collaborative Innovation, Center of Quantum Matter, School of Physics, Peking University, Beijing, China; ^4^State Key Laboratory for Metastable Materials Science and Technology, Yanshan University, Qinhuangdao 066004, China; ^5^School of Physical Sciences, University of Chinese Academy of Science, Beijing 100190, China; ^6^Beijing Key Laboratory for Nanomaterials and Nanodevices, Beijing 100190, China; ^7^QTF Centre of Excellence, Department of Applied Physics, Aalto University, FI-00076 Aalto, Finland; ^8^Songshan-Lake Materials Laboratory, Dongguan, 523808 Guangdong Province, China

## Abstract

Breaking the space-time symmetries in materials can markedly influence their electronic and optical properties. In 3R-stacked transition metal dichalcogenides, the explicitly broken inversion symmetry enables valley-contrasting Berry curvature and quantization of electronic angular momentum, providing an unprecedented platform for valleytronics. Here, we study the valley coherence of 3R WS_2_ large single-crystal with thicknesses ranging from monolayer to octalayer at room temperature. Our measurements demonstrate that both A and B excitons possess robust and thickness-independent valley coherence. The valley coherence of direct A (B) excitons can reach 0.742 (0.653) with excitation conditions on resonance with it. Such giant and thickness-independent valley coherence of large single-crystal 3R WS_2_ at room temperature would provide a firm foundation for quantum manipulation of the valley degree of freedom and practical application of valleytronics.

## 1. Introduction

Valley degree of freedom known as valley pseudospin, the local degenerate energy extrema in momentum space, can open up new ways to encode and process binary information: valleytronics [1–3]. Due to the intrinsic inversion symmetry breaking and threefold rotational symmetry, monolayer transition metal dichalcogenides (TMDCs), where a pair of degenerate direct bands locate at the corners of the Brillouin zone [4, 5], provide perfect playgrounds for valleytronics [6–11]. However, the extremely low carrier mobility and poor valley response at room temperature are significant roadblocks to the valleytronic applications with monolayer TMDCs [6, 12, 13]. Therefore, it is of the utmost importance to explore TMDCs with robust valley phenomena and high carrier mobility at room temperature.

**Figure 1:**
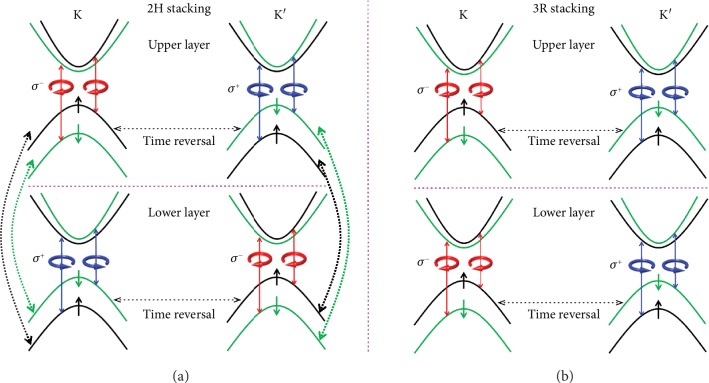
Symmetry-dependent valley physics. (a) Schematics of band structures and optical transition selection rules in 2H-bilayer TMDCs. Dashed arrows that connected the same spin between the upper and lower layers indicate interlayer hopping. (b) Schematics of band structures and optical transition selection rules in bilayer TMDCs with 3R stacking order. Spin configurations are indicated by ↑ (spin up) and ↓ (spin down). *σ*^−^ (*σ*^+^) denotes left (right) circularly polarized (circular arrows).

Few-layer TMDCs with a much higher mobility than monolayer may be an unprecedented venue for valleytronics since the carrier mobility increases with the increasing number of layers [12, 14]. For TMDCs, there are two distinct semiconducting crystal structures that originated from the different stacking orders, *i.e.*, 2H and 3R stacking [13, 15]. The different lattice symmetries between 2H and 3R phases result in contrasted valley properties, as described below in the case of bilayer. In the 2H-stacked bilayer TMDCs, the lower layer is a *π* in-plane rotation of the upper layer, which leads to the inversion symmetry restore. Therefore, topological valley physics governed by the global symmetry is prohibited [16], verified by both theoretical calculation [17] and experimental valley Hall effect [18]. Although highly selective circular polarization was observed in different 2H-stacked bilayer TMDCs [18–21], such circular dichroism stems from the hidden spin polarization and cannot signify valley polarization [17, 22, 23], which can be clearly seen in the diagram of the electronic structure (Figure 1(a)). The layer rotation symmetry switches the K and K′ valleys but leaves spin unchanged. The interband transitions in both the K (K′) valley of the upper layer and K′ (K) valley of the lower layer couple to *σ*^−^ (*σ*^+^) circularly polarized light (Figure 1(a)). Under circularly polarized radiation, such as *σ*^+^, both the K′ valley of the upper layer and K valley of the lower layer would be excited simultaneously, giving rise to the zero valley polarization and coherence [17, 22]. As a consequence, few-layer TMDCs with 2H symmetry are not a good playground for valleytronic applications.

In marked contrast, 3R-stacked TMDCs with the layers retaining the same orientation possess broken inversion symmetry [13, 24–26]. Figure 1(b) presents the schematics of band structures and optical transition selection rules in bilayer 3R-TMDCs. The interband transitions of the K (K′) valley in both upper and lower layers exclusively couple to *σ*^−^ (*σ*^+^) circularly polarized light. Thus, 3R-stacked TMDCs preserve the same valley-contrasting Berry curvature and physical properties as the case of monolayer, demonstrated recently in 3R MoS_2_ [13] and WS_2_ spiral nanostructures [27] where the inversion symmetry is broken. Accordingly, 3R-stacked few-layer TMDCs provide an unprecedented candidate for valleytronics and quantum logics [13, 28–30].

In addition to the well-known valley polarization at cryogenic temperature [6, 10, 31], an important step to test the valley index as a potential information carrier is to demonstrate the coherent manipulation of arbitrary valley states with linearly polarized laser light at room temperature [32, 33]. Moreover, valley-dependent optical selection rules are the necessary conditions for the generation of valley coherence. As a consequence, valley coherence is usually less than valley polarization and can reflect the quality and uniformity of samples better [33]. In this paper, we demonstrate the giant valley coherence in 3R-stacked WS_2_ large single-crystal at room temperature. Our measurements show that the direct excitons possess robust and thickness-independent valley coherence up to 0.742, establishing a firm basis for the manipulation of exotic valley degrees of freedom.

## 2. Results

**Figure 2:**
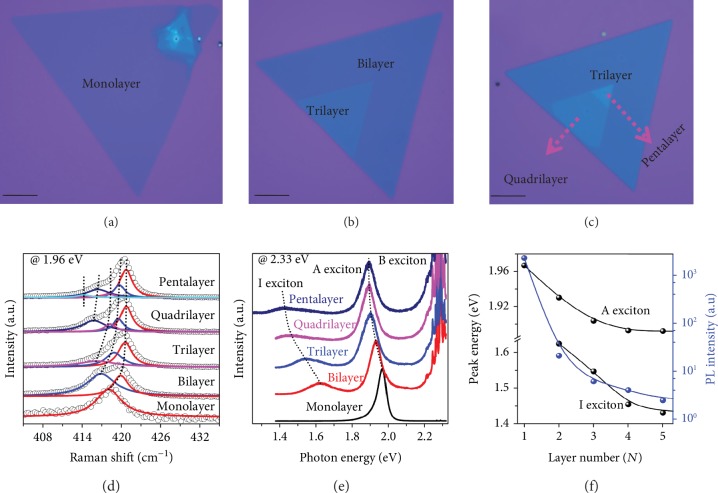
Microscopy characterizations of 3R WS_2_ with distinct thicknesses. (a–c) Optical micrograph of representative 3R WS_2_ samples with different layers (1L-5L). Scale bar: 20 *μ*m. (d) Raman spectra under 1.96 eV excitation, on resonance with the A exciton. Lorentzian fitting of the A_1g_(*Γ*) phonon modes is shown. (e) Normalized PL spectra by the intensity of the A exciton. The spectra were taken under the same conditions using 2.33 eV excitation. (f) Peak positions of excitons and integrated PL intensity as a function of layer numbers.

Since natural bulk crystal TMDCs including MoS_2_, WS_2_, MoSe_2_, and WSe_2_ are usually 2H phase [34], it is difficult to obtain multilayer with 3R symmetry through mechanical exfoliation. In this work, 3R-stacked WS_2_ layers were obtained by a chemical vapor deposition (CVD) method [35], using sulfur (S) and tungsten trioxide (WO_3_) powder as precursors (described in Materials and Methods). Figures 2(a)–2(c) show the white-light micrographs of representative samples with different thicknesses. The domain size can be larger than 100 *μ*m for monolayer, bilayer, and trilayer samples. The number of layers is first visually identified by observing their interference color through the optical microscope and later confirmed by Raman and photoluminescence (PL) spectroscopy.  Figure 2(d) displays the evolution of Raman spectra versus the number of layers, excited by 1.96 eV radiation on resonance with the A exciton. From the Lorentzian fitting, it can be seen clearly that the A_1g_(*Γ*) mode of WS_2_ splits into *N* components for *N* layers, in good agreement with previous results [35, 36].  Figure 2(e) presents the thickness-dependent normalized PL results. The PL spectra of bilayer and multilayer display three emission peaks corresponding to indirect band-gap exciton I (lowest energy) and direct-gap transition A (intermediate value) and B (highest energy), respectively. Note that the sharp peaks around the B excitons stem from the phonon modes, while PL spectrum of monolayer WS_2_ consists of only a single narrow feature (A exciton), indicating that monolayer is a direct-gap semiconductor. The absence of B exciton is due to the fact that the energy of B exciton in monolayer is larger than excitation photon energy (2.33 eV) [37].  Figure 2(f) shows the peak positions and integrated PL intensities as a function of layer numbers. With increasing the thickness, the energies of excitons are softened and PL intensities dramatically drop.

**Figure 3:**
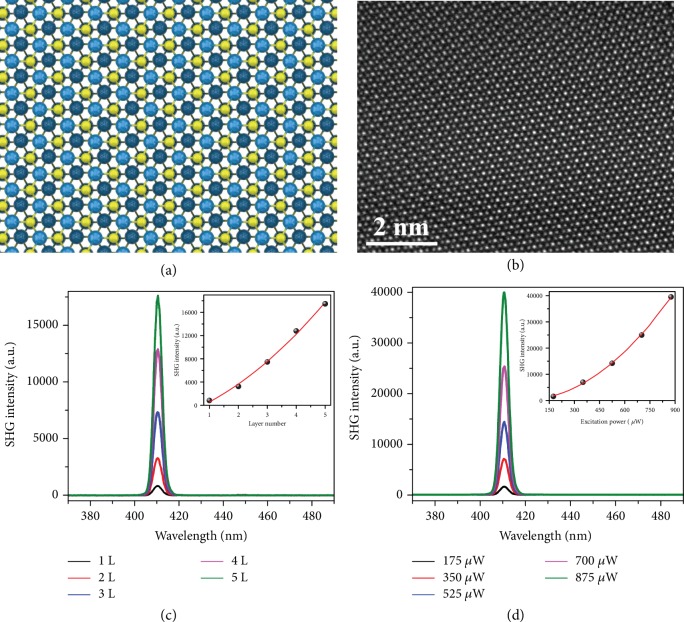
Symmetry and structural characterizations. (a) Top view of the stick-and-ball lattice structure of trilayer 3R WS_2_. The blue and yellow spheres represent W and S atoms, respectively. (b) Atomic resolution ADF-STEM image of 3R-stacked trilayer WS_2_. (c, d) Layer-dependent (c) and power-dependent (d) SHG spectra of WS_2_ with 3R stacking under excitation of *λ*_ex_ = 820 nm. The insets in (c) and (d) show the parabolic increase of the SHG intensity with increasing the number of layer and power density, respectively.

It can be clearly seen that the layers in few-layer WS_2_ samples possess the same orientation (Figures 2(b) and 2(c)). This indicates that our WS_2_ samples should be 3R phase [38, 39]. The stacking order of WS_2_ samples is further examined through aberration-corrected annular dark-field scanning transmission electron microscopy (ADF-STEM) and second harmonic generation (SHG). Since the layers in the 3R stacking samples maintain the same orientation but shift along the in-plane direction, the tungsten atoms locate at not only the corners of honeycomb lattices but also the center of hexagonal lattices when the number of layers is ≥3 (Figure 3(a)) [24]. This is in marked contrast to 2H phase that transition metal atoms of a given layer are sitting exactly on top of the chalcogen atoms of its neighboring layer and the metal atoms are only at the corners of hexagonal lattices. Figure 3(b) is the atomic resolution ADF-STEM image of our trilayer WS_2_ sample. Note that only tungsten atoms are observed since the contrast is proportional to the square of the atomic number. ADF-STEM image demonstrates that tungsten atoms sit at both the corners and center of honeycomb lattices, in fair agreement with the top view of trilayer WS_2_ lattice with 3R symmetry (Figure 3(a)).

Optical SHG, arising from the second-order nonlinear susceptibility tensor, is known as a sensitive probe to the crystalline inversion symmetry [40–42]. In stark contrast to the 2H-stacked TMDCs in which SHG intensity shows an even-odd oscillation with a decay envelope [41, 43], the efficiency of SHG from 3R-stacked TMDCs displays a quadratic dependence on the number of layers as a result of atomically phase-matched in-plane electric dipoles [24, 25]. Figure 3(c) presents the layer-dependent SHG spectra of our WS_2_ samples under excitation wavelength of *λ*_ex_ = 820 nm. The SHG intensity scales quadratically with the number of layers (inset in Figure 3(c)), in good harmony with 3R-stacked TMDCs that harbor broken inversion symmetry [25, 26]. The power-dependent SHG spectra of trilayer 3R WS_2_ are presented in Figure 3(d). The SHG intensity quadratically increases with the excitation power (inset in Figure 3(d)), in good agreement with the nonlinear optical principle [40].

**Figure 4:**
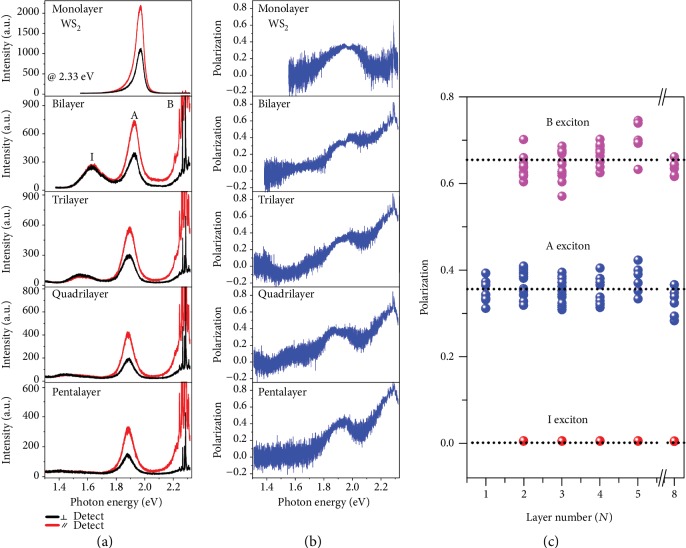
Layer-independent valley coherence under 2.33 eV excitation on resonance with the B exciton. (a) Linear-polarization-resolved PL spectra of 3R-stacked WS_2_ from monolayer to pentalayer at room temperature. Red and black curves present copolarized configuration (incident light polarization *e*_i_ and scattered light polarization *e*_s_ are parallel to each other) and crosspolarized (incident light polarization and scattered light polarization are perpendicular to each other), respectively. Sharp peaks around B excitons are Raman peaks from the WS_2_ and Si substrate. (b) Degree of valley coherence calculated from polarization-resolved PL spectra in (a). (c) Layer number (*N*)-dependent degree of linear polarization for B (magenta symbols), A (blue symbols), and I (red symbols) excitons. The distinct values within the same thickness are originated from different samples.

As the symmetry analysis above, 3R-stacked few-layer WS_2_ with inversion asymmetry may provide a perfect venue for quantum valleytronics. Now, we measure the valley physical properties of 3R-stacked WS_2_ with distinct thicknesses at room temperature. Since valley coherence is the optically generated quantum coherent superpositions of valley polarized excitons at K and K′ valleys and can well characterize the valley quality [9, 33, 44], we carried out the valley coherence measurement here. For each thickness, we measured at least six samples which exhibit almost the same valley phenomenon.  Figure 4(a) shows the linearly polarized PL spectra of representative 3R-stacked WS_2_ samples under a linearly polarized excitation of 2.33 eV at room temperature and two polarization configurations: copolarized (scattered and incident light are parallel to each other, *e*_i_||*e*_s_) and crosspolarized (scattered light is perpendicular to incident light, *e*_i_⊥*e*_s_). It can be seen unequivocally that I excitons have equal PL intensity for either copolarized or crosspolarized detection. In marked contrast, both A and B excitons display a pronounced linear polarization following the excitation. We quantify the valley coherence by the degree of linear polarization [9, 20]:

(1) $$\begin{array}{c} \rho =\frac{I_{\text{co}}-I_{\text{cr}}}{I_{\text{co}}+I_{\text{cr}}}, \end{array}$$

where *I*_co_ (*I*_cr_) is the PL intensity of copolarized (crosspolarized) configuration.  Figure 4(b) shows the degree of linear polarization calculated from polarization-resolved PL spectra in Figure 4(a) with Equation (1). The degree of linear polarization reaches the extremum when photon energy coincides with either A exciton or B exciton.

Figure 4(c) presents the evolution of valley coherence as a function of the layer number under 2.33 eV excitation. The distinct date points are obtained from different samples. The degree of linear polarization for I excitons is null. In stark contrast, both A and B excitons display prominent valley coherence that is almost independent on the number of layers, in good harmony with previous analyses that 3R-stacked few-layer TMDC samples possess the same valley physics with monolayer limit. The linear-polarization-resolved PL spectra of the 8-layer WS_2_ sample refer to Supplementary Materials ([Supplementary-material supplementary-material-1]). Average valley coherence for A and B excitons is 0.355 and 0.653, respectively, indicated by the dashed horizontal lines in Figure 4(c). We speculate that the larger valley coherence for B exciton is due to the fact that the 2.33 eV excitation is on resonance with the B exciton.

**Figure 5:**
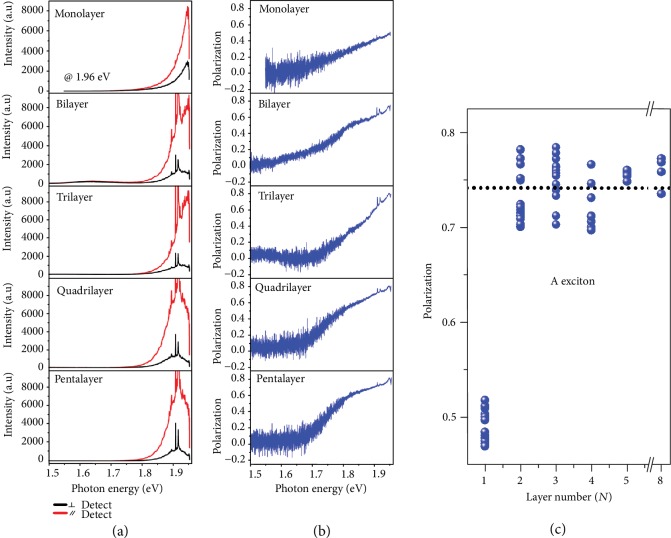
Layer-independent valley coherence under 1.96 eV excitation on resonance with the A exciton. (a) Linear-polarization-resolved PL spectra of 3R-stacked WS_2_ from monolayer to pentalayer at room temperature. Red (black) curve presents copolarized (crosspolarized) configuration. Sharp peaks around A excitons are Raman peaks from the WS_2_ and Si substrate. (b) Degree of linear polarization calculated from polarization-resolved PL spectra in (a). (c) The evolution of valley coherence for A exciton as a function of layer number (*N*). The distinct values within the same thickness are originated from different samples.

If the larger valley coherence for B exciton, excited by 2.33 eV radiation, is indeed caused by the fact that the excitation light is on resonance with it, the degree of linear polarization for A exciton will be greatly increased when the excitation photon energy is on resonance with it. We tune the energy of linearly polarized excitation light to 1.96 eV which is near the A exciton.  Figure 5(a) presents the linearly polarized PL spectra of representative monolayer and multilayer 3R WS_2_ samples recorded at room temperature, with the red (black) curve denoting copolarized (crosspolarized) detection configuration.  Figure 5(b) shows the corresponding degree of linear polarization determined from Equation (1) and polarization-resolved PL intensities in Figure 5(a). As the energy of emission photon approaches the A exciton, valley coherence increases monotonically and reaches the maximum when emission photon coincides with the A exciton.

Figure 5(c) shows the thickness-dependent degree of linear polarization under 1.96 eV excitation. Compared with 2.33 eV excitation, valley coherence of A exciton is strongly enhanced with 1.96 eV excitation. Except for monolayer, the valley coherence of A exciton under 1.96 eV excitation is 0.742 which is more than twice that excited by 2.33 eV radiation. The lower valley coherence of A exciton for monolayer is due to the fact that the energy of A exciton in monolayer is larger than excitation energy (Figure 2(f)).

## 3. Discussion

In conclusion, we have observed the valley physics of noncentrosymmetric 3R-stacked WS_2_ large single-crystal at room temperature by linear-polarization-resolved PL spectra. 3R-stacked WS_2_ possess robust and layer-independent valley coherence up to 0.742 at room temperature. The giant and thickness-independent valley coherence for multilayer 3R WS_2_ will push forward the development and application of valley quantum logics and optovalleytronic devices based on two-dimensional crystals with inversion asymmetry.

## 4. Materials and Methods

### 4.1. Chemical Vapor Deposition

Triangular WS_2_ domains were grown on the Si/SiO_2_ (300 nm) substrates in a home-made furnace with a 50 cm diameter quartz tube. 1.5 g sulfur (Alfa Aesar, purity 99.99%) powder was contained with a corundum boat and put at the upstream of the quartz tube. The distance from the corundum boat to the zone I center is 20 cm, and 2 g WO_3_ (Alfa Aesar, purity 99.8%) was loaded in another corundum boat and put into the zone II center. Subsequently, ultrahigh purity argon gas (35 sccm) was introduced into the quartz tube. The furnace was heated to 600°C (zone I) and 950°C (zone II) rapidly; in this case, S, WO_3_, and Si/SiO_2_ were kept at 120, 950, and 900°C, respectively. After 50 min, the furnace was cooled down to room temperature naturally.

### 4.2. Raman and PL Spectroscopy

The Raman and PL spectra were acquired in ambient conditions using a micro-Raman spectrometer (Horiba LabRAM HR Evolution) in a confocal backscattering configuration (confocal pinhole of 100 *μ*m). Laser power on the sample during Raman measurement was kept below 100 *μ*W in order to avoid sample damage and excessive heating. The backscattered signal was collected by an Olympus 100x objective lens and dispersed by a 600 g/mm grating for PL measurement and a 1800 g/mm grating to achieve Raman spectral resolution better than 1 cm^−1^.

### 4.3. Scanning Transmission Electron Microscopy (STEM) Characterization

The atomic resolution STEM characterizations were acquired from an aberration-corrected JEOL ARM300F transmission electron microscope which was operated at 80 kV with a convergence angle at 18 mrad and collection angles at 54~220 mrad.

## Supplementary Materials

Supplementary Materials S1: Raman spectra excited by 2.33 eV. S2: atomic resolution ADF-STEM image. S3: valley coherence of 8L WS_2_. S4: valley polarization.
