# Supplementary material for: Giant Valley Coherence at Room Temperature in 3R WS2 with Broken Inversion Symmetry
Source: Research (Wash D C). 2019 Oct 13;2019:6494565. doi: 10.34133/2019/6494565 (PMC6946257; doi:10.34133/2019/6494565)
Supplement: Supplementary Materials — S1: Raman spectra excited by 2.33 eV. S2: atomic resolution ADF-STEM image. S3: valley coherence of 8L WS2. S4: valley polarization. [file 6494565.f1.docx]

**Giant valley coherence at room temperature in 3R WS_2_ with broken inversion symmetry**

Luojun Du, Jian Tang, Jing Liang, Mengzhou Liao, Zhiyan Jia, Qinghua Zhang, Yanchong Zhao, Rong Yang, Dongxia Shi, Lin Gu, Jianyong Xiang, Kaihui Liu, Zhipei Sun, and Guangyu Zhang

**S1. Raman spectra excited by 2.33 eV**

Figure S1 is the Raman spectra with 2.33 eV excitation. We can see clearly the A_1g_(Γ), E1 2g(Γ) and 2LA(M) modes. Owing to interlayer interaction, the out-of-plane A_1g_(Γ) mode stiffens with increasing the number of layers.





**Fig. S1.** Raman spectra under 2.33 eV excitation

**S2. Atomic resolution ADF-STEM image**

**
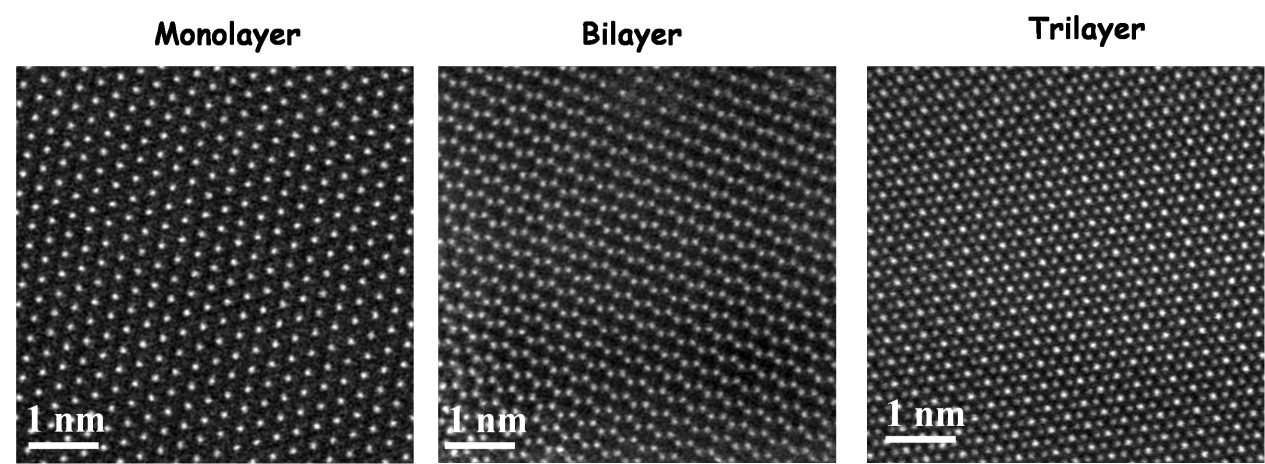
**

**Fig. S2.** Atomic resolution ADF-STEM image of 3R stacked monolayer, bilayer and trilayer WS_2_.

**S3. Valley coherence of 8L WS_2_**

Figure S3(a) is the optical micrograph of representative 3R WS_2_ samples with multilayer (ML). Figure S3(b) shows the SHG spectra of ML 3R WS_2_, as compared with 1L and 5L. It shows that the SHG intensity of ML 3R WS_2_ is about 62.7 (2.52) times that of 1L (5L), indicating that the number of layer is 8 for ML 3R WS_2_. Figure S3(c) and (d) present the linear-polarization-resolved PL spectra of 8L 3R WS_2_ under 2.33 and 1.96 eV excitation, respectively. The valley coherence of 8L 3R WS_2_ is akin to the results of 1L-5L in the main text.

**
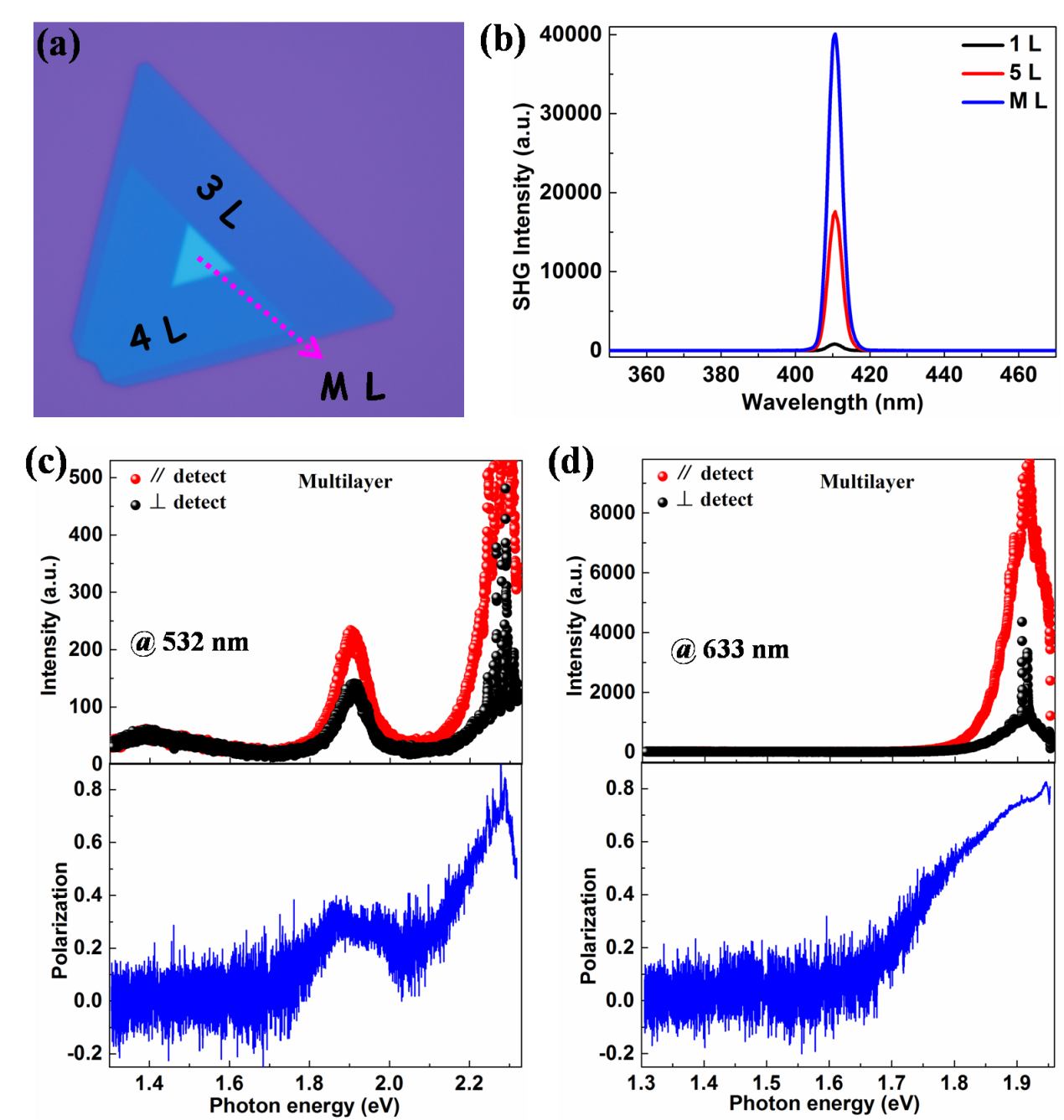
**

**Fig. S3. (a)** Optical micrograph of representative 3R WS_2_ samples with 3L, 4L and multilayer. **(b)** SHG spectra of 3R WS_2_ samples with 1L, 5L and multilayer. **(c)** Linear-polarization-resolved PL spectra of multilayer 3R WS_2_ using 2.33 eV excitation. **(d)** Linear-polarization-resolved PL spectra of multilayer 3R stacked WS_2_ using 1.96 eV excitation.

**S4. Valley polarization**

Figure S4 is the σ^+^ (black) and σ^-^ (red) resolved PL spectra for monolayer (Figure S4a) and bilayer (Figure S4b) 3R-WS_2_ at room temperature, excited by σ^+^ radiation with energy of 2.33 eV. The PL follows the helicity of the circularly polarized excitation. We quantify the degree of valley polarization as ρ = , where I(σ^±^) is the intensity of the left- (right-) handed circular-polarization component. The valley polarization of both the monolayer and bilayer 3R-WS_2_ is about 0.4 and slightly larger than the valley coherence (0.355) under the same condition.

**
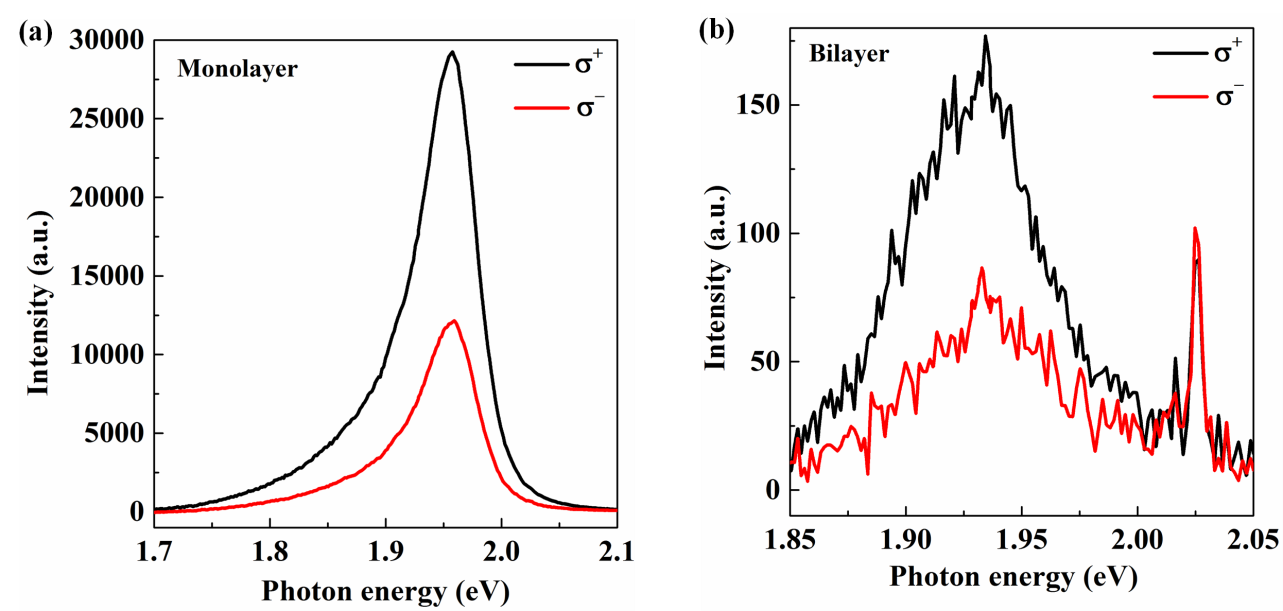
**

Fig. S4. Helicity-resolved PL spectra for monolayer (a) and bilayer (b) 3R-WS_2_ at room temperature.
